# Cu nanoparticles grafting on the surface of ZnO nanostructures to boost the porosity and surface area for effective removal of manganese ions from aqueous solutions

**DOI:** 10.1007/s11356-024-32625-2

**Published:** 2024-03-04

**Authors:** Ramadan A. Geioushy, Eman S. Ali, Ridha Djellabi, Mohamed A. Abdel-Khalek, Osama A. Fouad

**Affiliations:** 1 Central Metallurgical Research and Development Institute, P.O. Box 87, Helwan, 11421 Cairo Egypt; 2 Petrochemical Department, Egyptian Petroleum Research Institute, EPRI, Nasr City, Postal Code 11727 Cairo Egypt; 3 Departament d′Enginyeria Química, Universitat Rovira I Virgili, Av Països Catalans 26, 43007 Tarragona, Spain

**Keywords:** Cu grafted ZnO, Adsorption, Heavy metals, Mesoporous adsorbent

## Abstract

Designing highly adsorptive materials for wastewater treatment via facile approaches is still challenging. To boost the recovery of heavy metals from wastewater, surface and structure modification are considered a successful route. Herein, we report the design of ZnO nanoparticles by a simple thermal decomposition method followed by grafting Cu nanoparticles (Cu NPs) over the ZnO surface. Cu/ZnO was prepared with different Cu ratios, 0.01 and 1%. It was found that incorporating Cu into ZnO improved the porosity and surface area of ZnO. The adsorption ability of Cu/ZnO compared with bare ZnO was studied towards removing manganese ions from wastewater. The effects of several parameters, such as pH, temperature, contact time, and initial ion concentrations, were studied. The maximum removal of manganese was found at pH 2, 20 °C after 60 min in the presence of 1 g/L adsorbent. The role of Cu grafted on the surface of ZnO was discussed. The rates of adsorption were found to follow the pseudo-second-order model. The results showed better fitting to Freundlich isotherm. The thermodynamic study revealed that the sorption process is spontaneous, exothermic, and favorable at low temperatures. The free energy (ΔG°), enthalpy (ΔH°), and entropy (ΔS°) changes were calculated to predict the nature of adsorption.

**Graphical Abstract:**

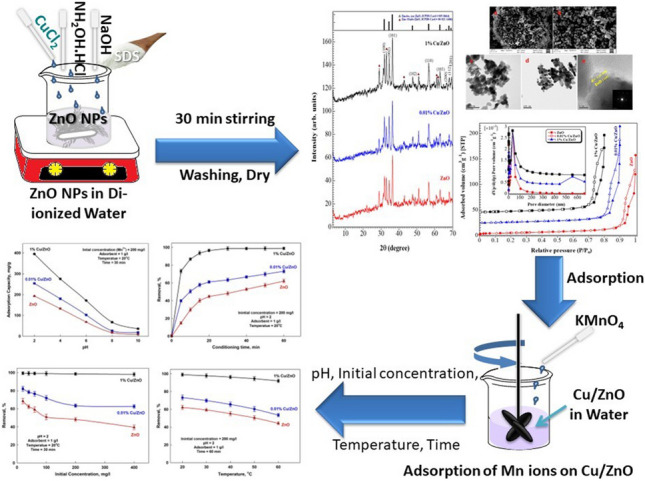

## Introduction

One of the consequences of the rapid growth in industry and human population is water contamination and infection, which subsequently increases the demand for fresh water. The number of societies living in water scare zones will increase to about 3.9 billion by 2030, as reported by the World Water Council (Xu et al. 2018). Sustainable human society development by mitigation of environmental pollution and health hazards is crucially related to wastewater management and potable water purification (Velusamy et al. 2021). The discharge of massive volumes of untreated industrial and wastewater containing organic, inorganic, and bioderived residues in water resources leads to the exhaustion of clean water. It might cause water-originated diseases and severe toxicological implications on the environment with a dramatic influence on human health. Notably, due to the non-biodegradable behavior, the accumulation of heavy metal ions such as manganese (Mn), chromium (Cr), copper (Cu), nickel (Ni), silver (Ag), lead (Pb), and cadmium (Cd) in the human body could damage the liver, kidney, brain function, nervous system, or even lead to death (Le et al. 2019).

In this regard, wastewater treatment becomes very important for sustainable development. The discharges from various industries such as textile, paper, dye, paint, and pharmaceutical are considered significant sources of organic, inorganic, and biological pollutants introduced into the natural water resources. The textile industry is generating significant quantities worldwide of wastewater polluted with heavy metal ions inclusion in dye molecules (Velusamy et al. 2021; De Gisi et al. 2016).

Various techniques have been developed to mitigate these risks by removing heavy metal ions from industrial wastewater. Some common methods include chemical precipitation, ion exchange, coagulation, reverse osmosis, electrolysis, membrane filtration, and adsorption (Adjeroud et al. 2018; Mirbagheri and Hosseini 2005; Ewecharoen et al. 2009; Zewail and Yousef 2015; Hebbar et al. 2016; Zheng et al. 2016). These techniques can be applied cooperatively and exhibit cons and pros in terms of efficiency, cost, and affinity toward a targeted treatment. Adsorption is a safe, effective, and low-cost technique for removing different pollutants from water (Gu et al. 2020). In addition, the adsorbent could be reused efficiently several times to remove pollutants (organic/inorganic) from wastewater. Searching for low-cost adsorbents has attracted attention other than costly polymeric materials used recently. Metal oxide nano adsorbents with small sizes and large surface areas have shown a significant performance for heavy metals removal (Saravanakumar et al. 2019). Zinc oxide nanoparticles (ZnO NPs) have been widely used in catalysis and gas sensor applications due to their unique properties, such as biocompatibility, low cost, and long-term stability. Recently, ZnO NPs have been used as adsorbents to remove heavy metal and organic pollutants from wastewater (Fouad et al. 2006; Rath et al. 2019). The applicability of ZnO NPs on large-scale applications is still limited due to their lower affinity towards some heavy metal ions, which might be related to the low surface area and non-functionality surface. Many strategies have been developed to improve the surface area of adsorbents and their capability. The increasing hydroxyl groups on the ZnO NPs surface reveal the significant potential in removing heavy metal ions (Yin et al. 2018). Moreover, the decoration of the ZnO surface with some metal/metal oxide NPs improves the surface area. It increases the number of active sites that enhance the adsorption activity of metal ions.

In this work, ZnO NPs were synthesized via a thermal decomposition route followed by Cu nanoparticles grafting on the ZnO surface. Incorporating Cu on the surface of ZnO can boost the porosity and functionality of ZnO, improving the fixation of heavy metals in water. The present report discusses the role of Cu grafted on ZnO towards the adsorption of Mn in water, wherein the Cu was incorporated with different ratios, 0.01 and 1%, on the surface of ZnO. The materials were characterized to check the effect of Cu on the surface, porosity, and functionality of ZnO. Sorption studies were carried out under different working conditions. The stability and recyclability of the adsorbent were studied. In addition, the adsorption mechanisms were investigated and discussed in depth.

## Experimental

### Materials

Zinc carbonate basic Laborchemie Apolda, copper chloride (≥ 99.5%, Sigma-Aldrich), sodium hydroxide (≥ 99.5%, Sigma-Aldrich), hydroxyl ammonium chloride (POCH ≥ 99.5%), and sodium dodecyl sulfate (SDS) were used without any purification. Potassium permanganate (KMnO_4_) of analytical grade, 99.9% purity (Sigma-Aldrich), was used for preparation of synthetic manganese ions (Mn^7+^) solution as (MnO_4_^−^) anions.

### Synthesis of Cu/ZnO nanoparticles

Firstly, ZnO was synthesized by a modified thermal decomposition technique, as mentioned previously (Hegazy et al. 2020). For Cu/ZnO preparation with different weight ratios, briefly, 0.1 g ZnO was dissolved in 18.1 mL deionized water, followed by the addition of a calculated amount of 0.1 M CuCl_2_ and 0.08 g SDS under vigorous stirring. After that, 1 M NaOH and 0.1 M NH_2_OH.HCl were added and stirred for 30 min. The white powder was collected and rinsed with deionized (DI) water and ethanol multiple times. Lastly, the samples were dried at 80 °C for about 16 h.

### Characterization of materials

X-ray diffractometer (XRD) D8Advance (Bruker), Germany, operated with Cu inner shell-K_α_ = 1.5406 Ǻ, was used for determining the phase structure. The surface’s morphology was inspected via Quanta FEG-250 field emission scanning electron microscope (FE-SEM) at 20 kV and JEOL-JEM-1230, Tokyo, Japan, high-resolution transmission electron microscope (HR-TEM). Jasco-V-770 UV/VIS/NIR spectrophotometer, Japan, integrated with VWBG-773-Band Gap Calculation software, carried out the absorption spectrum and bandgap estimation. The luminescence spectra were analyzed by means of a spectrofluorophotometer Shimadzu, RF-5301 PC (PL), Japan. X-ray photoelectron spectroscopy (XPS) analysis was undertaken by Al-Kα micro-focused mono-chromator XPS spectrometer, Thermo Scientific™ K-Alpha™ (up to 4 keV). The surface area analysis (BET) and N_2_ adsorption–desorption isotherms were collected at 273 k using Belsorb III equipment, Japan. The samples were degassed at 150 °C for 3 h before measurements. The manganese ion concentrations were determined by the colorimetric method. The absorbance was measured at λ = 515 nm, using a UV–visible spectrometer (PG Instruments T60).

### Adsorption experiments

Adsorption experiments were conducted using 0.05 g of absorbent with 50 mL of manganese solution (20–400 mg/L) at different pH and within the temperature range (20–60 °C) in a 100 mL round bottom flask. The flask was shaken at 200 rpm at the desired temperature in the water bath thermostat for a known time interval. The absorbent is removed from the solution using a centrifuge at 2000 rpm for 5 min.

The adsorption capacity of manganese at the end of the experiment is calculated using the following equation:

$$\mathrm{Adsorption\;capacity\;}{}^{{\prime}{\prime}}{q}^{{\prime}{\prime}} ({\text{mg}}/{\text{g}}) = \frac{\left(\mathrm{Ci }-\mathrm{ Cf}\right) \times V}{{\text{M}}}$$

where *C*_i_ and *C*_f_ are the initial and final concentrations of manganese ions (mg/L), *V* is the volume of the solution (L), and *M* is the mass of adsorbent (g).

The removal efficiency is calculated using the following equation (Hu et al. 2006):

$$\mathrm{Removal\;efficiency\;\% }= \frac{\mathrm{Ci }-\mathrm{Cf }}{{\text{Ci}}}\times 100$$

## Results and discussion

### Sample characterization

**Fig. 1:**
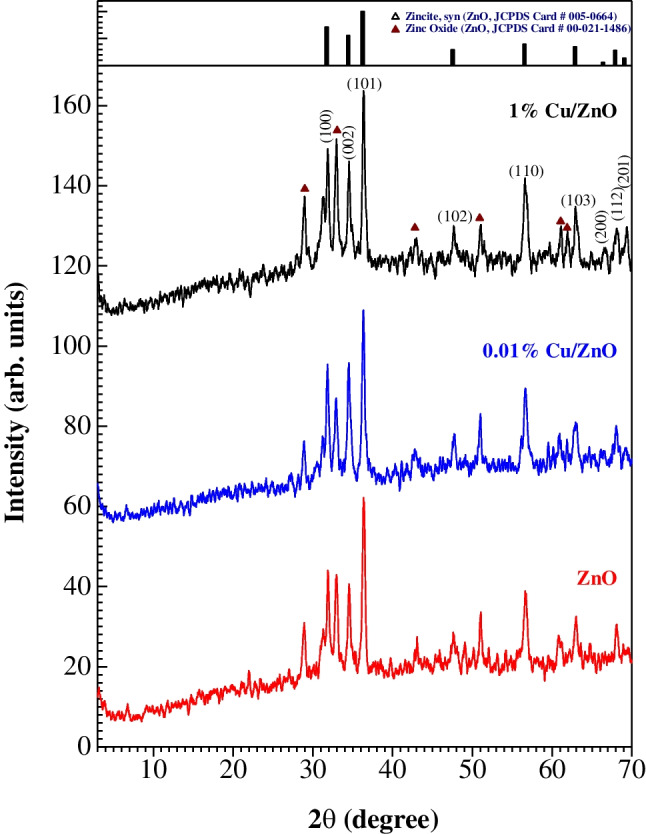
XRD patterns of the as-synthesized ZnO and Cu/ZnO NPs

Figure 1 shows the XRD diffraction patterns of ZnO, 0.01% Cu/ZnO, and 1% Cu/ZnO samples. Obviously, all diffraction peaks correspond to the ZnO phase (JCPDS cards no. 005–0664 and 00–021-1486). No peaks related to the copper phase appeared due to the low content of copper. The low peak intensity implies the small particle size of the synthesized ZnO.

**Fig. 2:**
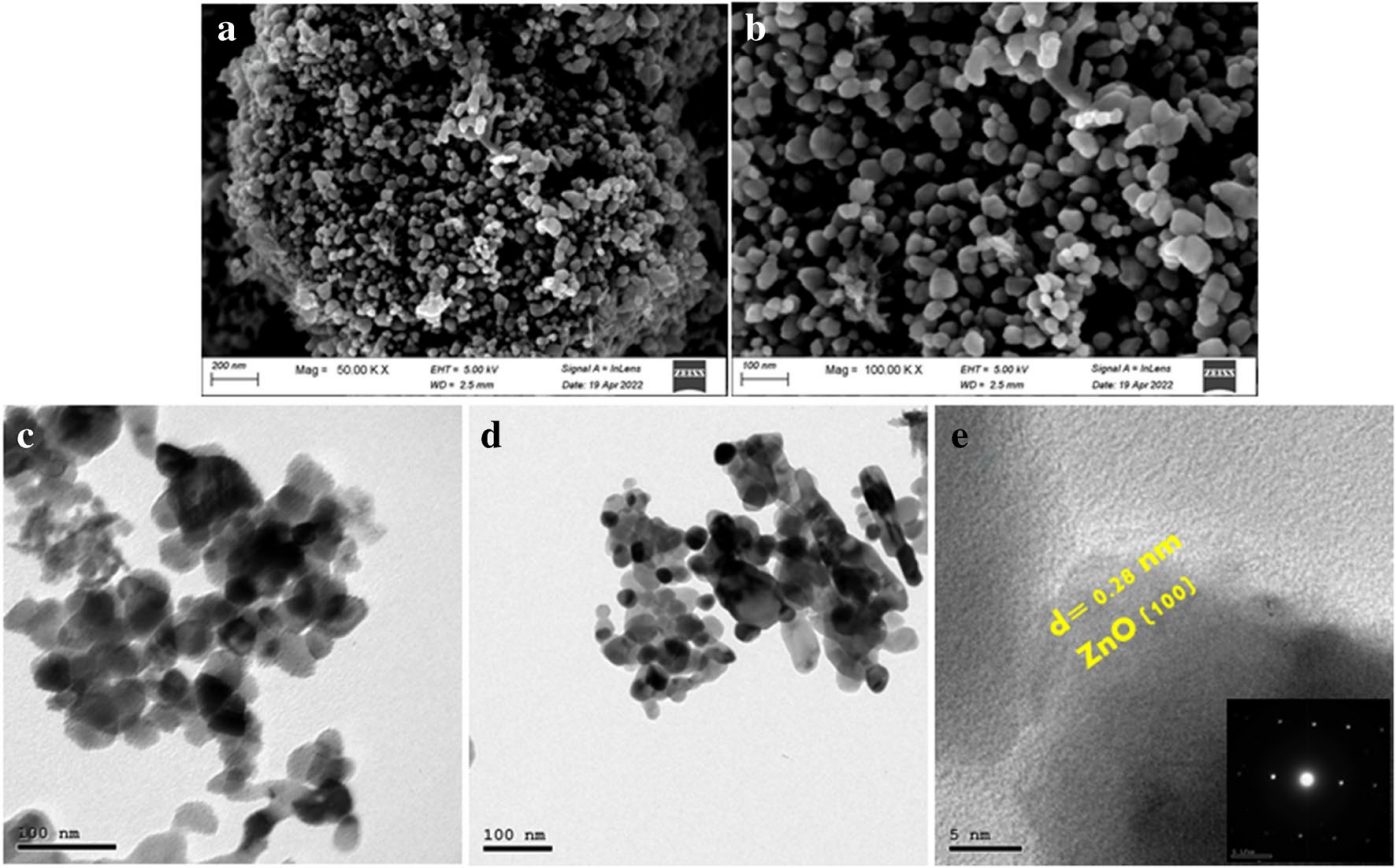
**(a–b)** SEM images and (**c–e)** TEM and HR-TEM images of 1% Cu/ZnO NPs sample

The morphology of the as-synthesized 1% Cu/ZnO sample was investigated by SEM and HR-TEM. It can be seen that ZnO is formed as spherical particles with an average size of about 25 nm, as shown in Fig. 2a and b. Moreover, TEM images confirmed the hexagonal structure of the ZnO phase, consistent with the obtained XRD data. The lattice fringe of a d-space value of 0.28 nm is related to ZnO’s (100) crystal plane (Geioushy et al. 2022). SAED pattern (inset Fig. 2e) indicates the single crystal of ZnO NPs.

**Fig. 3:**
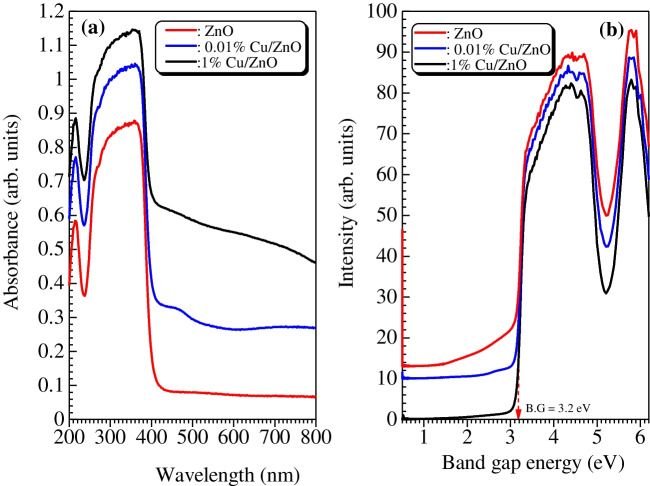
UV–vis absorption spectra (**a**) and band-gaps (**b**) of ZnO and Cu/ZnO NPs samples

**Fig. 4:**
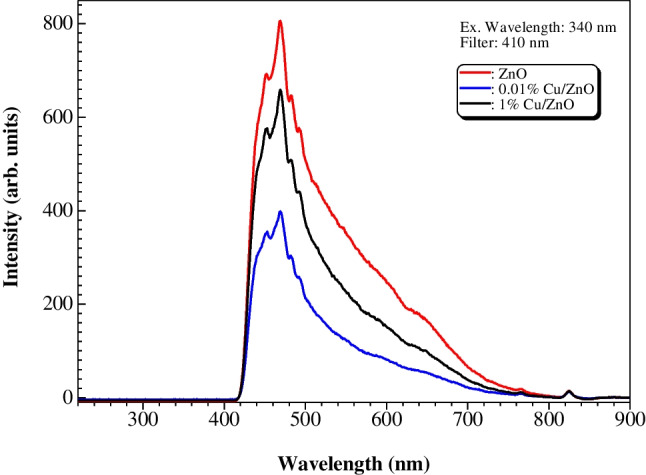
PL spectra of ZnO and Cu/ZnO samples at the excitation wavelength of 340 nm

The optical properties of the as-synthesized samples were measured using DRS and PL instruments. Figures 3a and b show the UV–vis absorbance spectra of all samples. ZnO NPs exhibit a characteristic absorption peak at around 360 nm. After the incorporation of Cu, the absorption extends to the visible region. The calculated band gap of ZnO is 3.2 eV, and there is no significant change in band gap values of 0.01–1% Cu/ZnO samples. This might be due to the very low content of Cu added. Moreover, the PL spectra at excitation wavelength 340 nm shown in Fig. 4 display a broad peak at 470 nm for all samples. However, the peak intensity tends to decrease with Cu’s incorporation. The 0.01% Cu/ZnO sample performed a lower intensity peak than 1% Cu/ZnO, which might be related to the agglomeration of Cu NPs over the ZnO surface as observed from SEM images. Accordingly, the 0.01% Cu/ZnO sample revealed excellent charge carrier separation and hence good catalytic activity compared to other samples.

**Fig. 5:**
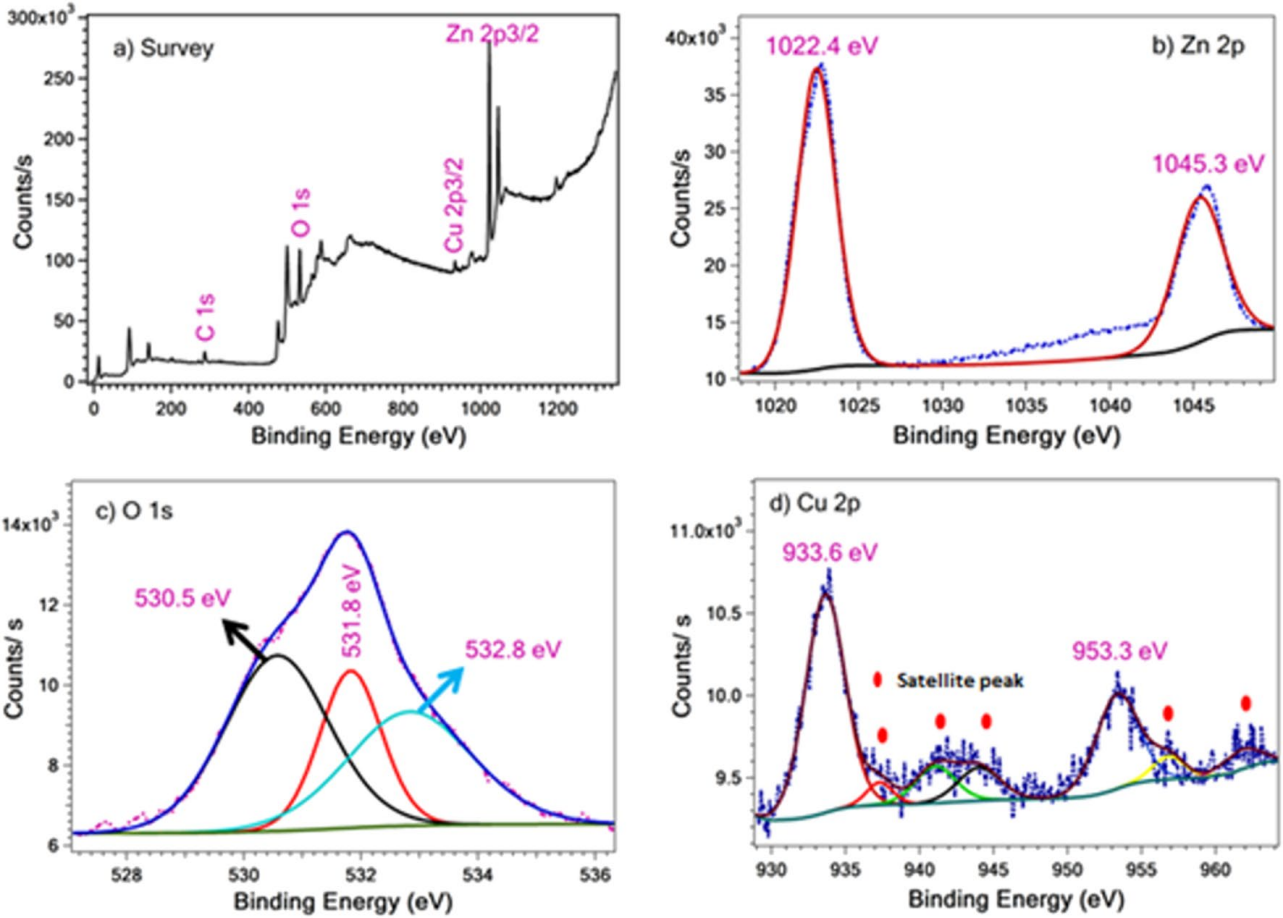
XPS spectra of 1% Cu/ZnO sample; (**a**) survey scan and high resolution of (**b**) Zn 2p, (**c)** O 1 s, and (**d**) Cu 2p

**Fig. 6:**
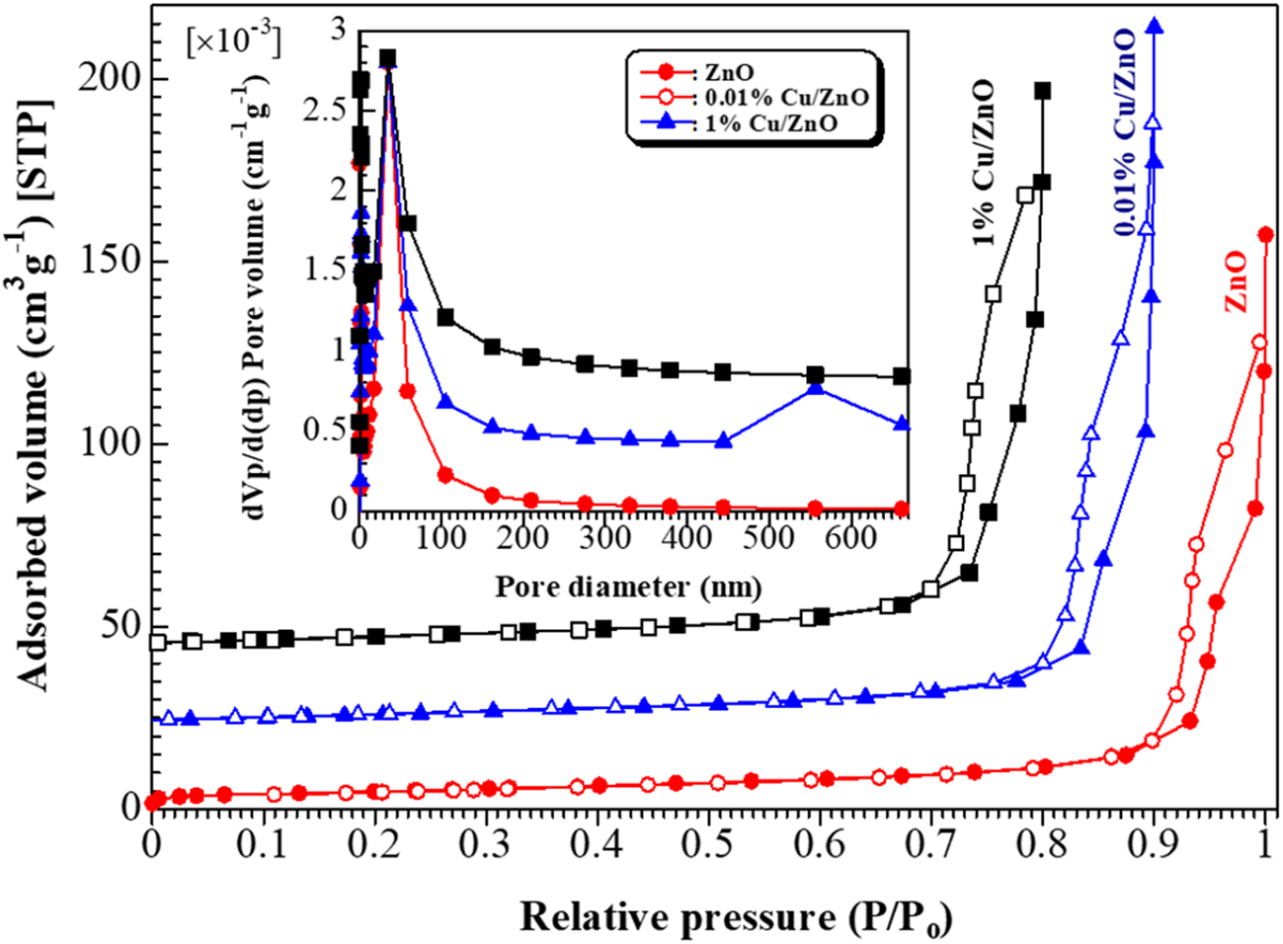
N_2_ adsorption/desorption isotherms for ZnO and Cu/ZnO, Inset is the pore size distribution

The chemical composition and oxidation state of the as-synthesized Cu/ZnO sample was investigated via XPS analysis, as shown in Fig. 5. The survey scan of 1% Cu/ZnO sample, Fig. 5a, confirmed that the existence of Zn, O, and Cu elements indicates the successful synthesis of Cu/ZnO nanostructure. Figure 5b shows the splitting of the Zn 2p peak in which the peaks at 1045.3 eV and 1022.4 eV corresponding to Zn 2p1/2 and Zn 2p3/2, respectively, which characteristics of Zn^2+^ ion reveal the Zn–O formation (Geioushy et al. 2017). The O 1 s splitting displays three binding energies peaks at 530.5, 531.8, and 532.8 eV, as shown in Fig. 5c. The peaks at 530.5 and 531.8 eV are characteristics of the Zn–O bond and OH, respectively (Geioushy et al. 2017; Liu et al. 2006). However, the peak at 532.8 eV is attributed to oxygen vacancies (Hegazy et al. 2020). The Cu 2p splitting spectrum shows the characteristic peaks of Cu 2p1/2 and Cu 2p3/2 at 953.3 and 933.6 eV, respectively. The intensity of Cu 2p peaks is low, and the splitting of these Cu 2p peaks indicates the trace amounts of the two oxidation states of Cu (I and II) (Fig. 5d) (Geioushy et al. 2017; Ma et al. 2015). The N_2_ adsorption/desorption isotherms were applied to determine the surface area and the porous structures of the as-prepared samples, as shown in Fig. 6. The isotherm of ZnO and Cu/ZnO samples displaying the same shape, which fit well with IV type, indicates the mesoporous structure of all samples. It is clear that with the incorporation of Cu, the surface area increased. The Cu/ZnO samples performed a higher surface area than the ZnO sample. Moreover, the Barrett-Joyner-Halenda (BJH) method estimated the pore size distribution, as shown in the inset of Fig. 6. The ZnO and 1% Cu/ZnO samples show only one peak. In contrast, the 0.01% Cu/ZnO sample show two kinds of pore size distribution. It is well known that these characteristics of large surface area and mesoporous structure play a significant role in enhancing the adsorption performance of Cu/ZnO nanostructures towards heavy metal ions (Lei et al. 2015).

### Adsorption study

#### Effect of contact time

**Fig. 7:**
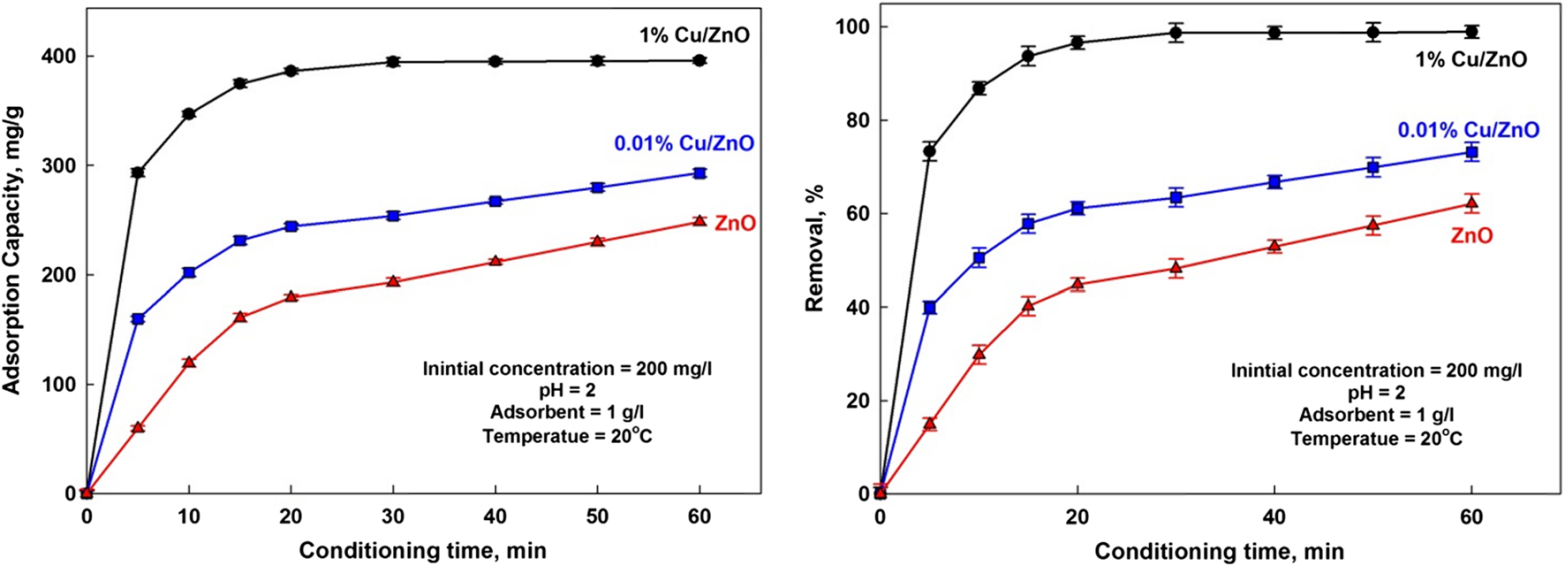
Effect of contact time on the adsorption capacity and removal efficiency

The uptake capacity of ions was investigated as a function of time to determine an optimum contact time for the adsorption. The effect of contact time on adsorption amount is shown in Fig. 7. The removal efficiency reached about 30, 50, and 87% for ZnO, 0.01% Cu/ZnO, and 1% Cu/ZnO, respectively, while it reached about 62, 73, and 99% for ZnO, 0.01% Cu/ZnO, and 1% Cu/ZnO after 60 min. When ions interacted with the adsorbent, the adsorption site on the solid surface gradually decreased. Still, the adsorption percentage depended on the number of ions transported from the bulk liquid phase to the actual adsorption site, so the adsorption percentage increased with time until saturation (Selim et al. 2020). The high adsorption in the first 10 min may be due to the gradual decrease in the adsorption sites on the solid surface. The amount of manganese ions transported from the bulk liquid phase to the solid surface controls the adsorption process. Therefore, the adsorption increased with time until saturation (Khalek et al. 2019). The maximum adsorption capacity of 1% Cu/ZnO is 396 mg/g for manganese ions, while it is 248 mg/g for ZnO.

#### Effect of pH

**Fig. 8:**
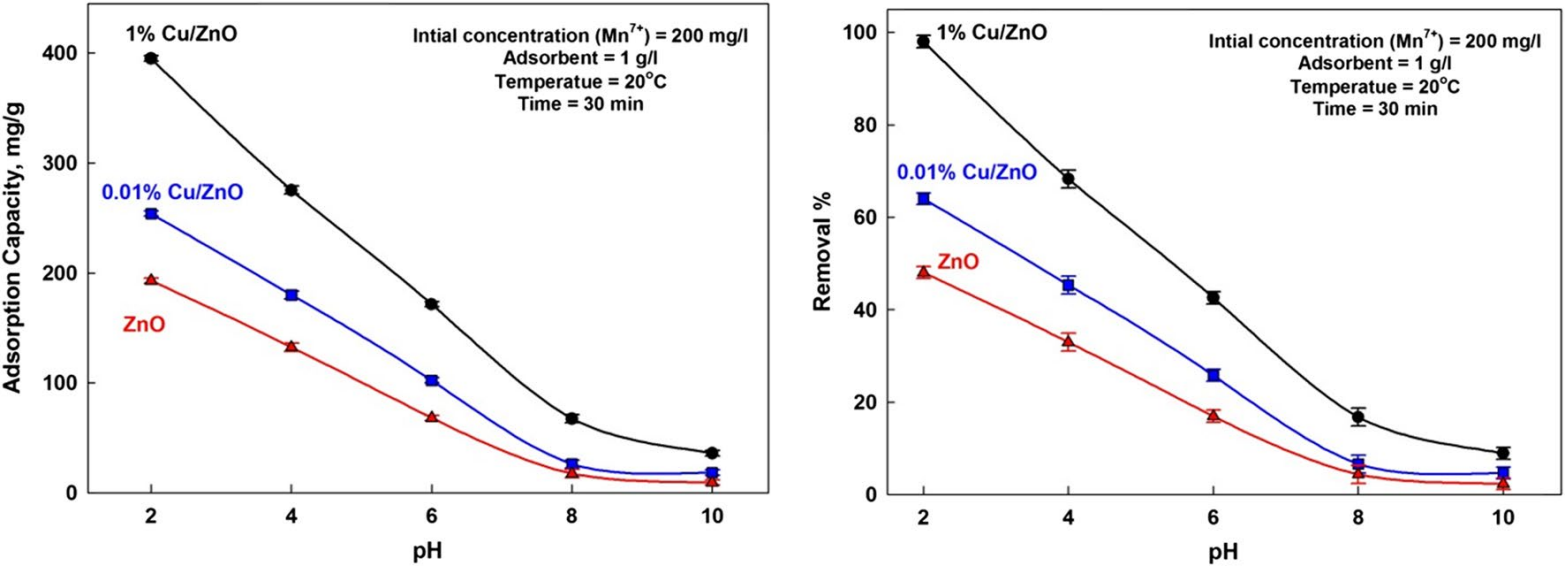
Effect of pH on the adsorption capacity and removal efficiency

Real wastewaters are discharged with different pH values; therefore, the evolution of the adsorptive ability of adsorbents is required to facilitate their transfer to real-world applications. pH can affect the adsorption process in different ways, such as modifying surface charge, the chemical behavior of metallic ions, and the electrostatic interaction between the adsorbent and the pollutant. Generally, the ionization of the adsorbent surface depends on its zero-point charge (Hassan et al. 2020; Abdel-Hameed et al. 2021; Abdulghany et al. 2021). Figure 8 indicates that the adsorption capacity and removal efficiency of manganese ions decreases with increasing the pH of the solution. The maximum removal of manganese ions is occurred at pH = 2. It is reached to 48%, 64%, and 98% for ZnO, 0.01% Cu/ZnO, and 1% Cu/ZnO, respectively. The maximum sorption capacity is 193, 254, and 395 mg/g for ZnO, 0.01% Cu/ZnO, and 1% Cu/ZnO, respectively.

This behavior might be due to electrostatic attraction between negative manganese anions (MnO_4_^−^) and the positive surface of the adsorbent. On the other hand, at higher pH values, pH ≥ 8, the adsorption of manganese ions reduced due to the precipitation of manganese hydroxide species. The manganese ions have different forms depending on the solution’s pH. Also, the activity of the adsorbent’s functional groups is strongly affected by solution pH (Abdulghany et al. 2021; Farahat et al. 2021; Sanad et al. 2021). Thus, the pH affects the sorption performance on the solid surface. In an acidic medium, the functional groups on the adsorbent surface are protonated, and the positively charged surface dominates. Thus, the attraction of the negatively charged (MnO_4_^−^) ions is more suitable in an acidic environment. De-protonation of the functional groups occurs in the alkaline medium, and these behave as negatively charged moieties, resulting in reduced attraction between negative species (Abdelbasir and Khalek 2022; Farahat et al. 2022).

#### Effect of initial ions concentration

**Fig. 9:**
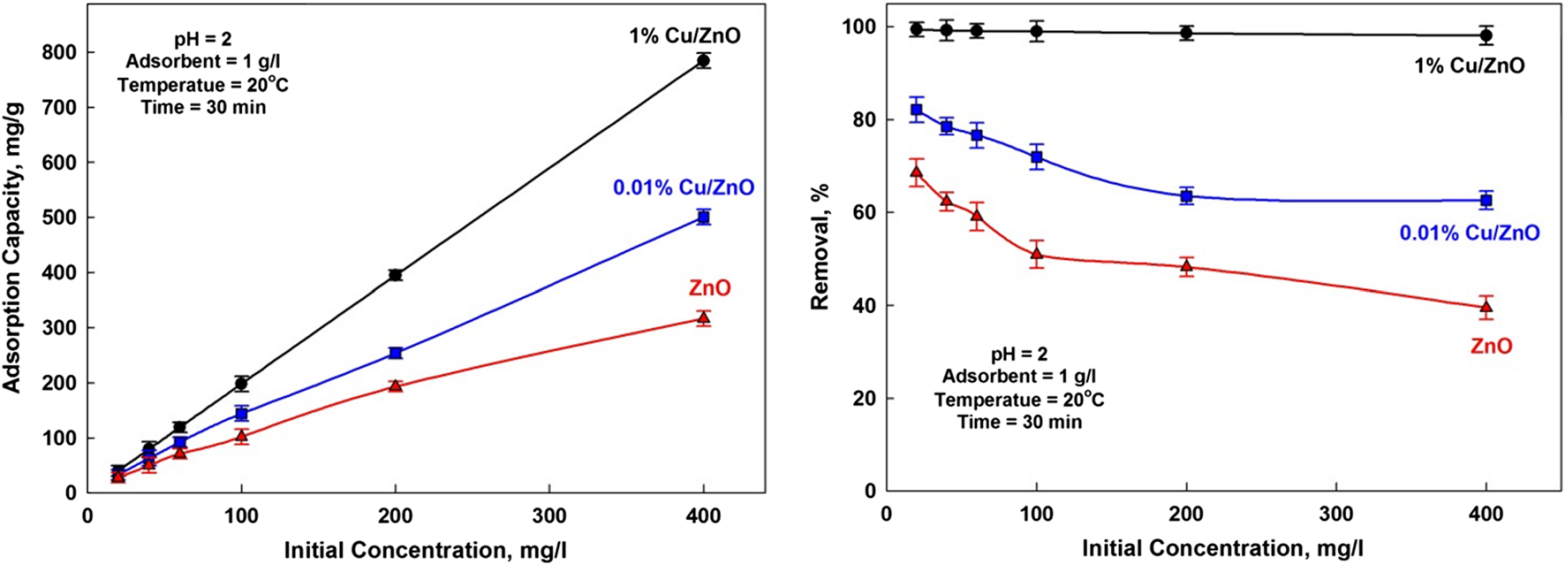
Effect of Mn^7+^ concentration on the adsorption capacity and removal efficiency

Initial ions concentration in aqueous solution significantly affects the adsorption process (Abdel-Khalek et al. 2017). The adsorption equilibrium concentrations (Fig. 9) shows that the adsorbed amount increased as the initial manganese concentration increased, and meanwhile, the removal rate decreased. A high initial concentration means that more ions are available, and thus, more ions are adsorbed for constant adsorbent mass (El-Hosiny et al. 2018; Khalek et al. 2019). At higher initial concentrations, the driving forces to overcome the mass transfer resistance for migration of ions from the bulk solution to the adsorbent solid surface increases (Mahmoud et al. 2019; Shehab et al. 2019). However, each unit mass of adsorbent is subjected to a larger number of ions, which gradually fill up the sites until saturation is reached. Increasing the initial concentration led to an increase in adsorption capacity and a decrease in removal efficiency. It is due to higher residual ions at higher concentration (Abdel-Khalek et al. 2020; Selim et al. 2020). It is worth mentioning that as the initial ion concentrations increased, the adsorption capacity increased, but the removal efficiency decreased due to higher residual ions at higher concentration. Also, as the amount of the adsorbent material increased, it is expected that the number of adsorbed ions would increase, and therefore the removal efficiency increased. But despite the increase in the adsorbed amount, it is attributed to the amount of adsorbent material added. As the amount of adsorbent material increased, the adsorption capacity (number of adsorbed ions per adsorbent material) decreased. These results are matched with that represented in Fig. 7 where the adsorption capacity is about 185 mg/g at the initial concentration of 200 mg/L in both figures.

#### Adsorption kinetics

The adsorption kinetics was observed at different contacting times. The initial concentration of ions in solution was fixed at 200 mg/L. To determine the uptake kinetic mechanisms of ions adsorbed. Two kinetic models were investigated: pseudo first-order and pseudo-second-order (Attia et al. 2022). Their linear equations are presented as follows:

The pseudo-first-order equation:

$$\mathrm{In}\;q_e-q_t=\mathrm{In}\;q_e-k_1t$$

The pseudo-second-order equation:

$$\frac{t}{{q}_{t}}= \frac{1}{{k}_{2}{q}_{e}^{2}}- \frac{t}{{q}_{e}}$$

where *q*_t_ (mg/g) and *q*_e_ (mg/g) are the number of ions adsorbed by the adsorbent at time t (min) and at equilibrium and *k*_1_ (min^−1^) and *k*_2_ (g·mg^−1^·min^−1^) are the equilibrium rate constants.

**Fig. 10:**
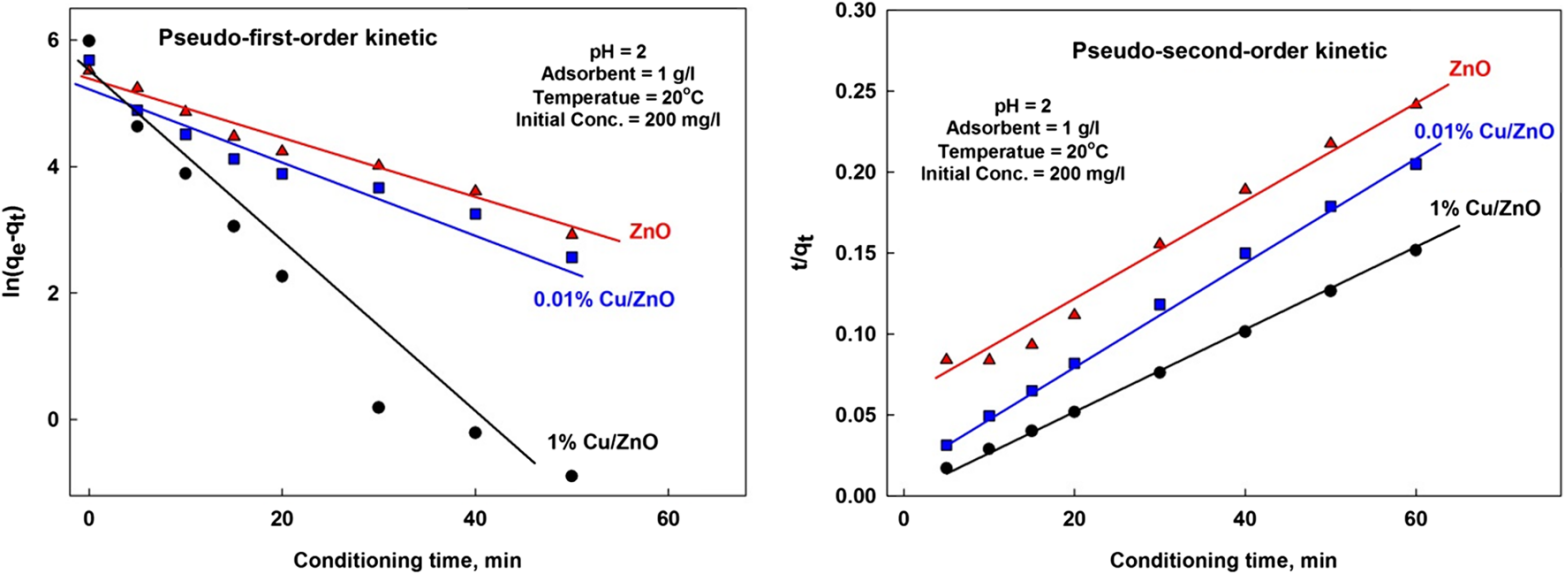
Plots of pseudo-first-order and pseudo-second-order models

Adsorption kinetics results of linearized pseudo-first-order and pseudo-second-order models obtained are shown in Fig. 10 and Table 1. On the view of the values of coefficient of determination *R*^2^, it was evident that pseudo-second-order model was the better in describing adsorption kinetics. Moreover, pseudo-second-order kinetic model predicts closer values of the maximum adsorption capacity with the experimental values, and hence it gives the applicability of this model.

**Table 1:** Adsorption kinetics parameters of pseudo-first-order and pseudo-second-order models

Parameter	1% Cu/ZnO	0.01% Cu/ZnO	ZnO
The pseudo-first-order parameters			
R^2^	0.9531	0.9339	0.9751
K_1_	− 0.1384	− 0.0537	− 0.0484
Calculated q_max_	201.3	183.2	218.6
Experimental q_max_	395.8	292.8	248.4
The pseudo-second-order parameters			
* R*^2^	0.9997	0.9976	0.9851
* K*_2_	1.68 × 10^−3^	3.21 × 10^−3^	3.36 × 10^−3^
Calculated q_max_	400.0	312.5	298.2
Experimental q_max_	395.8	292.8	248.4

#### Isotherm models study

Adsorption isotherm is significant for adsorption analysis behavior (Abdel-Khalek et al. 2017). Langmuir and Freundlich isotherms were used to investigate the adsorption process.

The Langmuir isotherm (El-Hosiny et al. 2018) can be expressed as follows:

$$\frac{{C}_{f}}{{q}_{t}}=\frac{{C}_{f}}{{q}_{max}}+ \frac{1}{{bq}_{max}}$$

The Freundlich isotherm (Khalek et al. 2019) can be written as follows:

$${ln\;q}_t=ln\;k+\frac1nln\;C_f$$

where *C*_f_ (mg/L) is the final ions concentration, *q*_t_ (mg/g) is the adsorbed amount at time t, *q*_max_ (mg/g) (maximum adsorption) is monolayer adsorption capacity, *b* (L/mg) is a binding constant related to the free energy of adsorption, *K* is the indicative of the extent of the adsorption, and *n* is the adsorption intensity.

**Fig. 11:**
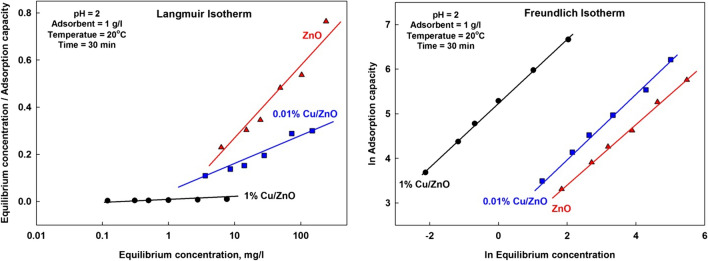
Langmuir and Freundlich adsorption isotherms for Mn^7+^ ions adsorption onto nanostructures

The adsorption isotherms are presented in Fig. 11 and Table 2. The adsorption was described by Langmuir isotherm model, which assumes that the adsorption occurs at a specific homogeneous surface of the adsorbent. That is, during the adsorption process, ions have to move through the pores and the channels of the lattice so that to replace the exchangeable ions of solid surface. The regression (*R*^2^) of Langmuir model linear fitting is 0.81 and 0.91, and it predicts maximum sorption capacity highly deviated from the experimental results. On the other hand, the regression (*R*^2^) of Freundlich model linear fitting is more than 0.996, and it predicts maximum sorption capacity closed to the experimental results. So, the adsorption results fit well by Freundlich model better than Langmuir model, which meant the adsorption process, is mainly physically adsorption (Mahmoud et al. 2019; Shehab et al. 2019).

**Table 2:** Equilibrium parameters for Langmuir and Freundlich isotherms

Parameter	1% Cu/ZnO	0.01% Cu/ZnO	ZnO
Langmuir isotherm parameters			
* R*^2^	0.9152	0.8189	0.9164
* q*_max_ (calculated)	1250	769	476
* q*_max_ (experimental)	785	500	316
* b*	0.2051	0.0094	0.0072
Freundlich isotherm parameters			
* R*^2^	0.9989	0.9963	0.9971
* n*	1.3900	1.4150	1.4806
* q*_max_ (calculated)	798	512	327
* q*_max_ (experimental)	785	500	316
* K*_F_	188.5	13.61	7.95

The effect of isotherm shape can also predict whether an adsorption system is “favorable” or “unfavorable.” The essential features of the Langmuir isotherm can be expressed in terms of a dimensionless constant separation factor or equilibrium parameter *R*_L_ which is defined by the following relationship (Abdel-Khalek et al. 2020).

$${R}_{L}=\frac{1}{1+b{C}_{i}}$$

where C_i_ (mg/L) is the initial ions concentration and *b* is the Langmuir constant. The value of *R*_L_ indicates the shape of the adsorption isotherm to be favorable or unfavorable. The value of *R*_L_ between 0 and 1 indicates favorable adsorption, while *R*_L_ = 1 suggests unfavorable adsorption, and the adsorption process is linear adsorption, while *R*_L_ = 0 represents irreversible adsorption.

Table 3 shows that the values of *R*_L_ are between 0 and 1, indicating a favorable and reversible adsorption process.

**Table 3:** The equilibrium parameter (*R*_L_) as a function of initial manganese concentration

Initial concentration	1% Cu/ZnO	0.01% Cu/ZnO	ZnO
	b = 0.2051	b = 0.0094	b = 0.0072
	R_L_		
20	0.9596	0.9981	0.9985
40	0.1086	0.7267	0.7764
60	0.0752	0.6394	0.6983
100	0.0465	0.5155	0.5814
200	0.0238	0.3472	0.4098
400	0.0120	0.2101	0.2577

#### Effect of temperature

**Fig. 12:**
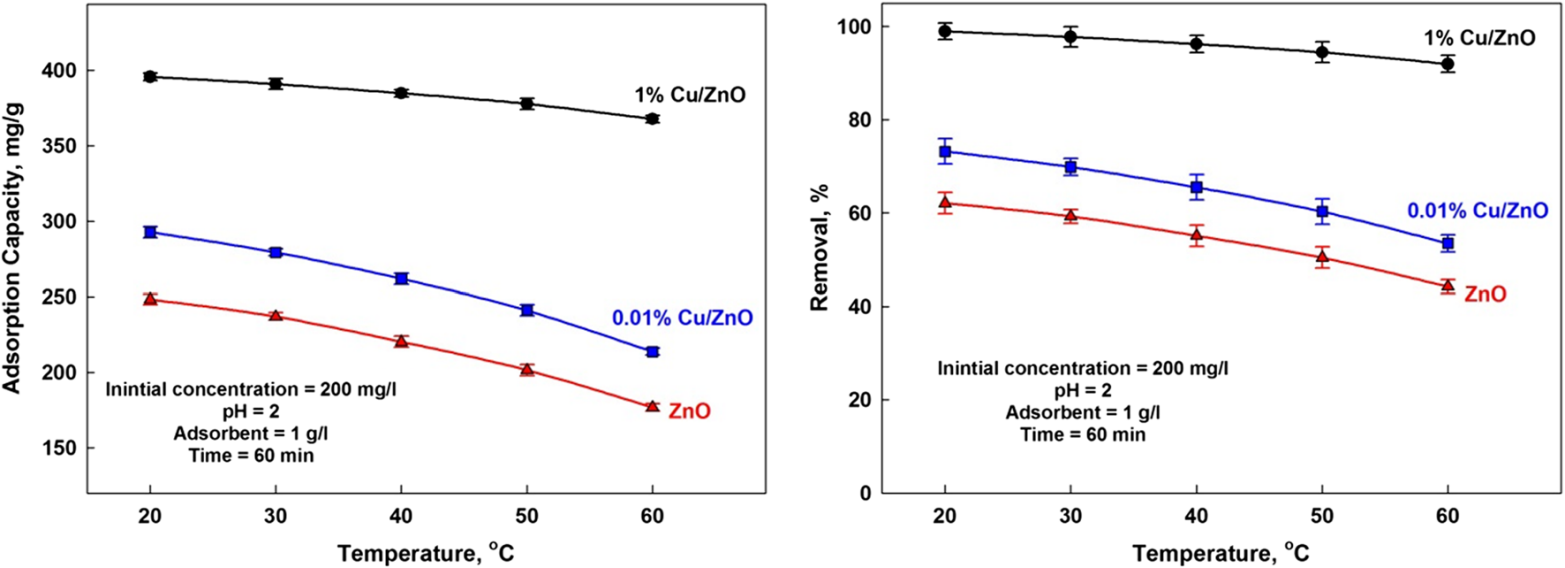
Effect of temperature on the adsorption capacity and removal efficiency

Figure 12 shows the sorption performance as a function of temperature, although it is known that the adsorption process is induced by increasing the temperature (El-Hosiny et al. 2018). In this case, the sorption decreased by increasing the temperature from 20 to 60 °C. A decrease in the sorption capacity with rising temperature may be due to the damage of active sorption sites of the composite surface. It may be due to an increasing the tendency to desorb species from the interface to the solution. These results suggest the exothermic nature of the sorption process onto the surface of ZnO composite (Attia et al. 2022).

#### Thermodynamic study

**Fig. 13:**
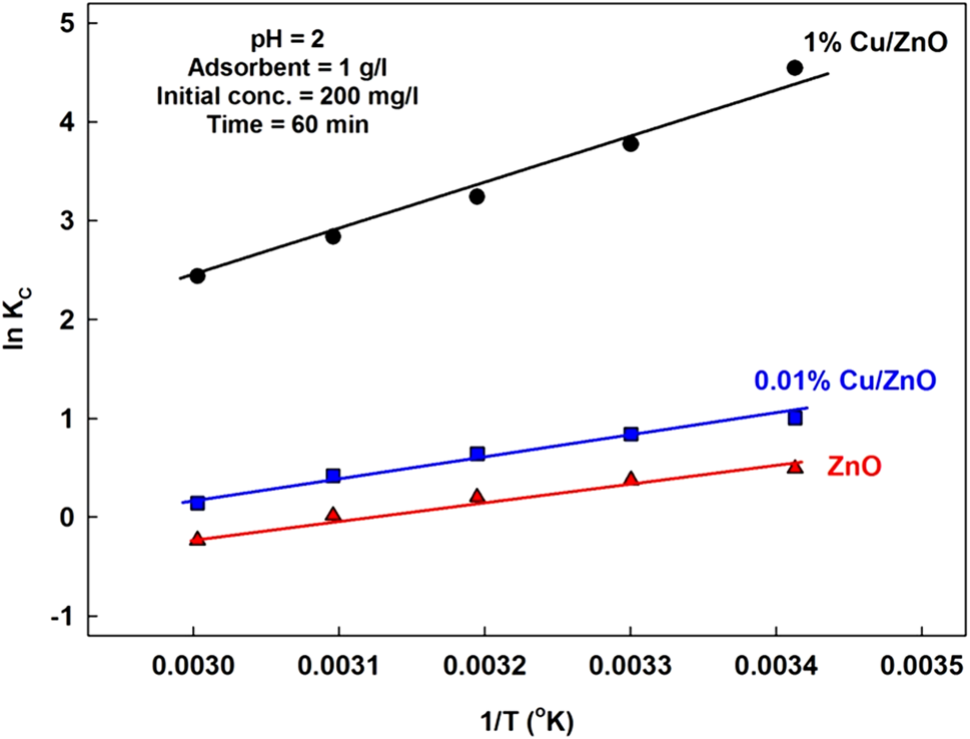
Plot of lnk_c_ versus 1/T

The spontaneity of the sorption process can be investigated through thermodynamic parameters such as enthalpy (ΔH°), entropy (ΔS°), and Gibb’s free energy (ΔG°). They were determined from the slope and intercept of the straight line of lnKc versus 1/T plot, Fig. 13, using Van’t Hoff equation (Shehab et al. 2019):

$${\text{ln}}\;{K}_{c}=\frac{{\Delta S}^{^\circ }}{R}-\frac{{\Delta H}^{^\circ }}{RT}$$

where *T* is the temperature in degree K, *R* is the gas constant [8.314 J/mol K]. k_c_ = F/(1 − F), F = (C_i_ – C_f_)/C_i_, [C_i_ initial concentration & C_f_ final concentration], *T* is the temperature in degree K, and *R* is the gas constant [8.314 J/mol K]. Table 4 presents the data used for calculations and the k_c_ values.

**Table 4:** Data used for calculations and the k_c_ values

Temp., °C	1/T (°K)	**CZ-1%**							
		C_f_	C_i_—C_f_	F	**k**_**c**_	lnk_c_	ΔH° KJ.mol^−1^	ΔS° J.k^−1^	ΔG° J.k^−1^.mol^−1^
20 30 40 50 60	3.413 × 10^−3^ 3.300 × 10^−3^ 3.194 × 10^−3^ 3.096 × 10^−3^ 3.003 × 10^−3^	2.10 4.48 7.52 11.06 16.07	197.9 195.5 192.4 188.9 183.9	0.9895 0.9776 0.9624 0.9447 0.9197	**94.23** **43.64** **25.59** **17.08** **11.44**	4.5458 3.7760 3.2424 2.8381 2.4376	− **38.0**	− **106.4**	− 11073 − 9512 − 8437 − 7621 − 6748
Temp., °C	1/T (°K)	**CZ-0.01%**							
		C_f_	C_i_—C_f_	F	**k**_**c**_	lnk_c_	ΔH° KJ.mol^−1^	ΔS° J.k^−1^	ΔG° J.k^−1^.mol^−1^
20 30 40 50 60	3.413 × 10^−3^ 3.300 × 10^−3^ 3.194 × 10^−3^ 3.096 × 10^−3^ 3.003 × 10^−3^	53.60 60.27 68.99 79.41 93.05	146.4 139.7 131.0 120.5 106.9	0.7320 0.6987 0.6550 0.6030 0.5348	**2.7313** **2.3184** **1.8990** **1.5186** **1.1494**	1.0048 0.8409 0.6413 0.4178 0.1392	− **17.3**	− **50.6**	− 2447 − 2118 − 1668 − 1121 − 385
Temp., °C	1/T (°K)	**Z1**							
		C_f_	C_i_—C_f_	F	**k**_**c**_	lnk_c_	ΔH° KJ.mol^−1^	ΔS° J.k^−1^	ΔG° J.k^−1^.mol^−1^
20 30 40 50 60	3.413 × 10^−3^ 3.300 × 10^−3^ 3.194 × 10^−3^ 3.096 × 10^−3^ 3.003 × 10^−3^	75.80 81.43 89.78 99.16 111.5	124.2 118.5 110.2 100.8 88.4	0.6210 0.5928 0.5511 0.5042 0.4423	**1.6385** **1.4558** **1.2275** **1.0169** **0.7931**	0.4938 0.3756 0.2050 0.0168 − 0.2318	− **14.5**	− **45.3**	− 1202 − 946 − 533 − 45 641

The standard Gibbs free energy change ΔG° is estimated using the following equation:

$${\Delta\;G^\circ = -RT lnK}_{c}$$

The negativity of free energy change decreased with increasing the temperature, indicating a decrease in the ions sorption with the increase in temperature (Table 5). The monolayer capacity for the solid system decreased with an increase in temperature. The values of ΔG° for all systems were negative at different temperatures, except at 60 °C for ZnO, which reflects the spontaneous behavior of the adsorption process. Generally, decreasing the ΔG° negativity with increasing temperature indicates that the adsorption process becomes less favorable at high temperatures (Attia et al. 2022).

**Table 5:** Thermodynamic parameters for manganese ions adsorption

Adsorbent	ΔH° (KJ.mol^−1^)	ΔS° (J.k^−1^ mol^−1^)	ΔG° (J.mol^−1^)				
			20 °C	30 °C	40 °C	50 °C	60 °C
ZnO	− **14.5**	− **45.3**	− − 1202	− 946	− 533	− 45	641
0.01% Cu/ZnO	− **17.3**	− **50.6**	− − 2447	− 2118	− 1668	− 1121	− 385
1% Cu/ZnO	− **38.0**	− **106.4**	− 11073	− 9512	− − 8437	− 7621	− 6748

Adsorption of ions was exothermic in nature as reflected in the Langmuir adsorption isotherm and also corroborated by the negative ΔH° values (Table 5). The lower ΔH° values < 40 kJ/mol confirms the physical sorption (Attia et al. 2022). The negative values of ΔS° indicate the decreasing of randomness at the solid/liquid interface during the adsorption. It suggests the adsorption is less favorable at higher temperatures.

## Conclusions

In this report, we demonstrated the enhancement of the adsorption ability of ZnO via its modification by grafting Cu NPs on the surface. Cu/ZnO nanostructure was synthesized via a thermal decomposition technique followed by a simple co-precipitation route. It was found that Cu can improve the physicochemical characteristics of ZnO, including the surface area and porosity. ZnO was modified with different ratios of Cu, namely 0.01 and 1%, and both showed improved adsorption of Mn in water. The as-synthesized samples were characterized via XRD, SEM, TEM, PL, UV-DRS, XPS, and BET surface area techniques. All adsorbent samples showed higher adsorption at pH 2 and decreased with pH increasing due to the electrostatic attraction as a function of solution pH. The Freundlich model provided the best linearity for all systems, suggesting physical adsorption. The pseudo-second-order model is better in describing the adsorption kinetics. The negative ΔH° values suggest the exothermic nature of the sorption system, while the negative ΔS° indicates the decreasing of a random degree at the interface layer between solid and liquid. The free energy indicated the spontaneous behavior of the sorption process at low temperatures. Overall, incorporating a small amount of Cu NPs on the surface of ZnO could lead to excellent adsorption capacity because of the enhanced surface area and affinity towards Mn ions.

## Data Availability

The original data that support the findings of this study are available upon request.
